# Tuberous sclerosis complex with ovarian endometrioma misdiagnosed as multiple metastases: a case report

**DOI:** 10.3389/fmed.2026.1819173

**Published:** 2026-05-19

**Authors:** Hao Tang, Shan Peng, Pengyu Meng, Siyu Wang, Xiaoming Wang, Fei Yang

**Affiliations:** 1 Department of Neurology, Affiliated Hospital of North Sichuan Medical College, North Sichuan Medical College, Nanchong, China; 2 Department of Medical Rehabilitation, The Second Clinical College of North Sichuan Medical College, Nanchong Central Hospital, Nanchong, China

**Keywords:** bone scintigraphy, diagnosis, diagnostic imaging, neurocutaneous syndromes, tuberous sclerosis complex

## Abstract

Tuberous sclerosis complex (TSC) is an autosomal dominant genetic and multi-spectrum disease, characterized by the development of benign hamartomas in multiple organ systems, which may be misdiagnosed as malignant neoplasms. We present the case of a TSC patient with a large ovarian endometrioma that showed heterogeneous enhancement on a computerized tomography (CT) scan, and Tc-99 m-methylene diphosphonate (^99m^Tc-MDP) bone scintigraphy revealed the possibility of bone metastases. She was initially misdiagnosed with a malignant ovarian tumor and multiple metastases, whereas the definitive diagnosis was established based on clinical diagnostic criteria related to TSC. Following multidisciplinary management, our patient’s general condition remains satisfactory. We aim to enhance the understanding of TSC by summarizing the clinical manifestations and imaging findings of our patient in order to reduce the rates of missed and misdiagnosed cases.

## 1 Introduction

Tuberous sclerosis complex (TSC) is an autosomal dominant genetic disease resulting from a pathogenic variant in *TSC1* or *TSC2*, characterized by the development of benign hamartomas in multiple organ systems. The incidence of TSC ranges from 1 in 6,000 to 1 in 10,000, affecting approximately 2 million people worldwide; moreover, two-thirds of patients do not have a family history of TSC because these cases are sporadic (1). Facial angiofibromas, seizures, and intellectual disability have historically been considered the hallmarks of TSC. However, given that it is a condition with a wide phenotypic spectrum, the rates of missed diagnosis and misdiagnosis in clinical practice are rather high. In addition to benign tumor growth, TSC may coexist with malignant neoplasms (2, 3). This overlap further complicates the differentiation between TSC and malignant tumors.

We present the case of a young patient with TSC and an ovarian endometrioma (chocolate cyst) that was initially misdiagnosed as multiple metastases because of abnormal findings on bone scintigraphy.

## 2 Case presentation

We present the case of a woman in her 20s who had suffered from generalized tonic–clonic epileptic seizures, having experienced a total of five episodes during the past 4 months, and was admitted to the Department of Neurology. She also exhibited mental and behavioral abnormalities during the interictal period of the epileptic seizures. She reported no specific family history or previous seizure history. The physical examination revealed multiple skin lesions on her face, which were formerly diagnosed as facial capillary hemangioma, without further examinations and treatment having been performed previously.

The baseline laboratory workup was within normal limits, except for anemia, carbohydrate antigen 125 (CA-125), and human epididymis epithelium-secreted protein (HE4) levels (Table 1). Electroencephalography showed multiple epileptic discharges in different brain regions. Head magnetic resonance imaging (MRI) revealed multiple abnormal signals without enhancement in the intracranial area, including several tiny nodules in the bilateral lateral ventricles, which were also observed on CT (Figure 1A). The results of a basic cerebrospinal fluid analysis were normal, and autoimmune encephalitis antibody tests were also negative, thereby ruling out autoimmune encephalitis.

**Table 1:** Erythrocyte indices and tumor marker results.

Category	Result	Reference range
RBC	4.03 × 10^12^/L	3.80–5.10 × 10^12^/L
Hb	83 g/L	115–150 g/L
Hct	0.294	0.350–0.450
MCV	72.80 fl	82.00–100.00 fl
MCH	20.70 pg	27.00–34.00 pg
MCHC	284.0 g/L	316.0–354.0 g/L
AFP	2.02 ug/L	0.0–7.0 ug/L
CEA	0.97 ug/L	0.00–5.00 ug/L
HE4	73.3 pmol/L	0.0–60.5 pmol/L
CA-125	202.0 U/mL	0.0–35.0 U/mL
CA19-9	23.60 U/mL	0.00–34.00 U/mL

RBC, red blood cell count; Hb, hemoglobin; Hct, hematocrit; MCV, mean corpuscular volume; MCH, mean corpuscular hemoglobin; MCHC, mean corpuscular hemoglobin concentration; AFP, alpha-fetoprotein; CEA, carcinoembryonic antigen; HE4, human epididymis epithelium-secreted protein; CA-125, carbohydrate antigen 125; CA19-9, carbohydrate antigen 19–9.

**Figure 1:**
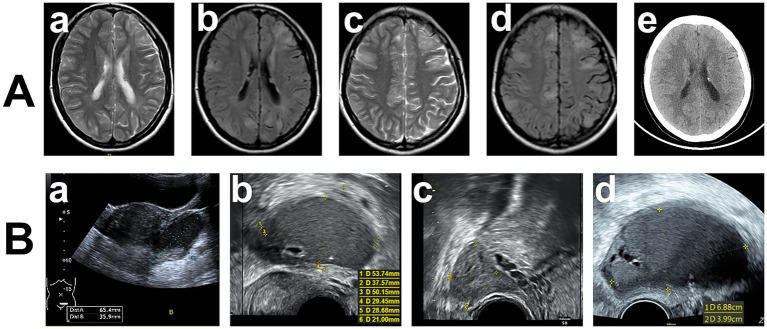
**(A)** a–d: Axial T2-weighted imaging (T2WI) and fluid-attenuated inversion recovery–magnetic resonance imaging (FLAIR MRI) showed multiple patchy hyperintense cortical tubers and tiny subependymal nodules. (d) Head CT showed tiny subependymal nodules. **(B)** a–c: Ultrasound images showed an ovarian endometrioma in the left adnexal region, with a size of approximately 6.8 × 4.0 cm. (d) The right adnexa showed no significant alterations at 3 months follow-up.

As the definitive diagnosis cannot be made solely based on the current symptoms and brain MRI findings, a preliminary assessment of the respiratory, cardiovascular, and digestive systems was conducted to screen for multisystem disease and avoid missed diagnoses, with special attention paid to the genitourinary system given the abnormal CA-125 and HE4 levels. Her abdominal ultrasound revealed a well-demarcated solid-cystic nodule with a size of approximately 6.8 × 4.0 cm in the left adnexal region, and the patient subsequently underwent transvaginal color Doppler ultrasound on the suggestion of the gynecologists (Figure 1B). The mass showed heterogeneous enhancement on contrast-enhanced CT; meanwhile, a left abdominal mass with heterogeneous enhancement was also identified (Figure 2). The chest and abdominal CT revealed multiple patchy high-density shadows in the vertebrae (Figure 3A). The detected adnexal and abdominal masses prompted us to consider the possibility of osteoblastic bone metastases. Therefore, bone scintigraphy was used to evaluate whether there was abnormal metabolic activity in the bones. ^99m^Tc-MDP bone scintigraphy revealed multiple foci of slightly increased uptake involving the cervical, thoracic, and lumbar spines, findings suggestive of possible osteoblastic metastases (Figure 3B). In light of the above findings, careful evaluation was performed about the possibility of malignant tumors in the adnexal region. Meanwhile, the patient agreed to undergo *TSC1/TSC2* genetic testing, which was negative.

**Figure 2:**
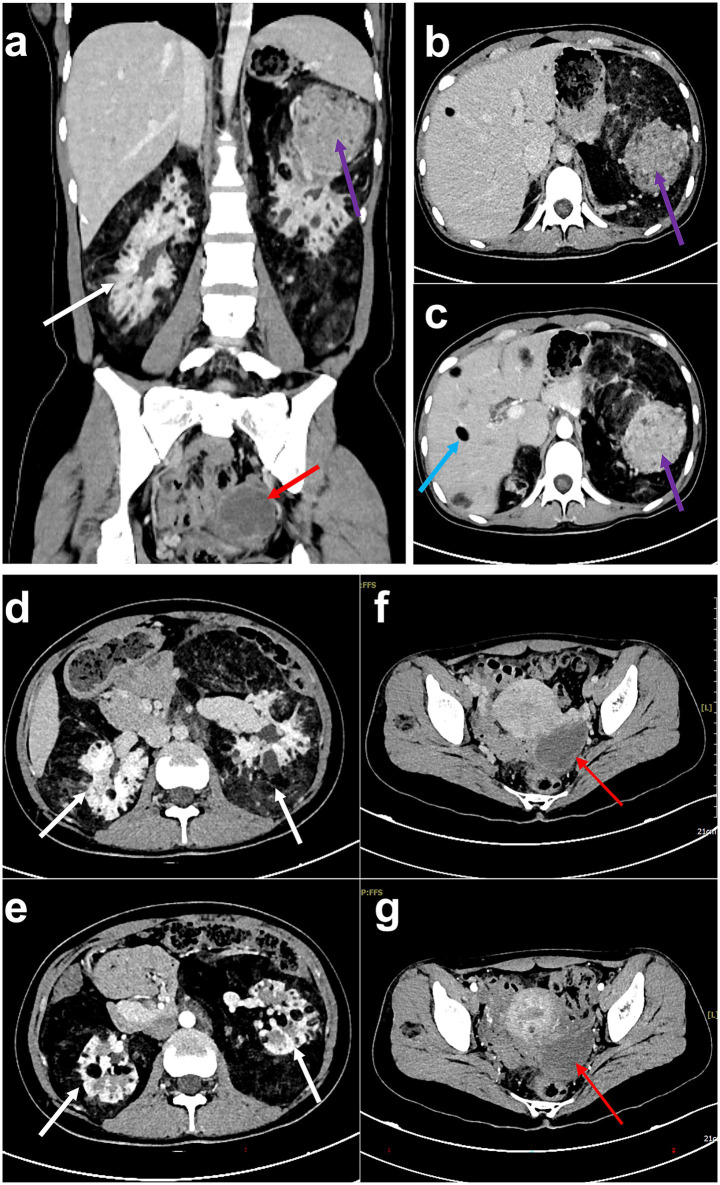
Abdominal CT revealed a regularly shaped mass in the upper abdomen originating from the left kidney with heterogeneous enhancement (purple arrows). The hepatic parenchyma contains multiple fat-attenuation nodules without obvious enhancement (blue arrow). The axial CT showed that the bilateral kidneys had enlargement and structural disorder and contained multiple fat density foci, without enhancement in the arterial phase (white arrows), and revealed a pelvic mass with smooth wall enhancement (red arrows).

**Figure 3:**
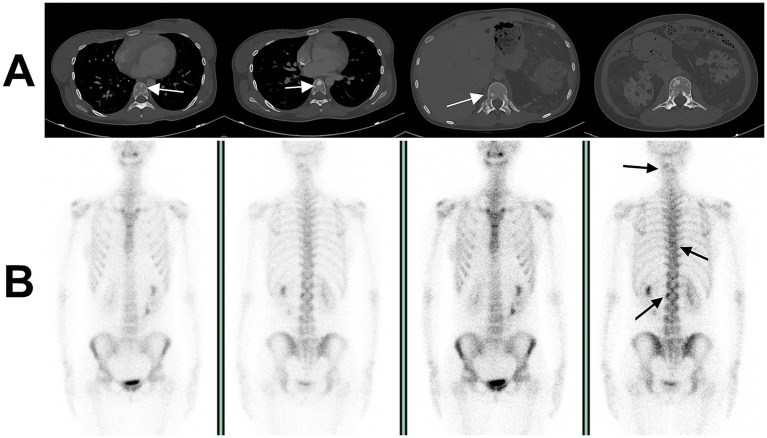
**(A)** The CT showed sclerotic bone diseases on vertebrae (white arrows). **(B)** The bone scintigraphy showed focal areas of increased radiotracer uptake in the cervical, thoracic, and lumbar spines (black arrows).

A multidisciplinary discussion was initiated to determine the nature of the pelvic mass, which was believed to be an ovarian endometrioma. On ultrasound, the ovarian mass appeared cystic with regular morphology and CT demonstrated smooth wall enhancement indicative of a benign lesion. Considering the patient’s history of dysmenorrhea, the pelvic mass was consistent with an endometriotic cyst, which could be a plausible explanation for the mild reduction in red blood cell count and mild elevation in CA-125 and HE4. In the evaluation of the abdominal mass, radiologists and oncologists collectively determined that the lesion originating from the left kidney had a regular morphology and did not demonstrate the typical imaging of increased bone density. The bone lesions were consistent with the diagnosis of sclerotic bone lesions (SBLs), as suggested by the absence of a primary malignant neoplasm. With multiple cortical tubers, angiomyolipoma, renal cysts, subependymal nodules, and SBLs present, the diagnosis was confirmed as TSC based on clinical criteria, even though genetic testing was negative.

The treatment was a maintenance regimen of levetiracetam to control seizures at a dose of 500 mg twice daily, along with donepezil 5 mg daily for cognitive impairment. The patient was hospitalized for 9 days and showed adequate control of seizures; then, she declined the subsequent surgical treatment. After 3 months, she underwent transvaginal color Doppler ultrasound again, which showed that there was no significant change in the ovarian endometrioma (Figure 1B). During follow-up for approximately 1 year, at intervals of approximately 6 months, she continued to be seizure-free with a normal routine hematological and biochemical profile.

## 3 Discussion

Mutations in the *TSC1* and *TSC2* tumor suppressor genes, located on chromosomes 9 and 16, respectively, are responsible for encoding proteins TSC1 and TSC2, which together form a protein complex that inhibits the mammalian target of rapamycin (mTOR) signaling pathway, and are regarded as an independent diagnostic criterion for TSC (4). Almost 10–15% of TSC cases may occur without a known mutation in *TSC1* or *TSC2* have been reported because of mosaicism, *de novo* mutation, or technically undetectable mutations (5). It is plausible that our patient has negative *TSC1/2* results in peripheral blood and no family history of TSC. Therefore, the absence of identifiable pathogenic variants does not rule out TSC. A clinical diagnosis of TSC could be made by either two major criteria or one major criterion with two minor criteria for a definitive diagnosis (Table 2) (6). The patient presented with facial angiofibromas, multiple cortical tubers, subependymal nodules, renal angiomyolipoma, multiple renal cysts, and SBLs, meeting four major clinical criteria and two minor criteria. Hence, the definitive diagnosis was established in the absence of identifiable pathogenic variants. As early and readily apparent clinical signs, skin features account for 4 of the 11 major criteria and 3 of the 7 minor criteria. Given that the onset of TSC manifestations varies significantly by age, the presence of related skin features should raise clinical suspicion—even if diagnostic criteria are not fully met—as exemplified by our patient.

**Table 2:** Diagnostic criteria for tuberous sclerosis complex.

Major criteria	Minor criteria
Hypomelanotic macules (≥3; at least 5 mm diameter)	“Confetti” skin lesions
Angiofibroma (≥3) or fibrous cephalic plaque	Dental enamel pits (≥3)
Ungual fibromas (≥2)	Intraoral fibromas (≥2)
Shagreen patch	Retinal achromic patch
Multiple retinal hamartomas	Multiple renal cysts
Multiple cortical tubers and/or radial migration lines	Non-renal hamartomas
Subependymal nodule (≥2)	Sclerotic bone lesions
Subependymal giant cell astrocytoma	
Cardiac rhabdomyoma	
Lymphangioleiomyomatosis	
Angiomyolipomas (≥2)	

Definite TSC: two major features or one major feature with two minor features. Possible TSC: either one major feature or two minor features.

Although the development of benign hamartomas is recognized as the hallmark of TSC, in rare instances, patients with TSC may develop malignant tumors. Renal cell carcinoma and pancreatic neuroendocrine tumor are regarded as the most common malignancies in patients with TSC (2, 3). *TSC1* plays a dual role in both suppressing tumors and promoting metastasis in the TGF-β-Smad pathway, which is crucial for cellular growth arrest and epithelial-mesenchymal transition, and works independently of *TSC2* (7). Therefore, it is currently believed that the occurrence rate of malignant tumors is more significant in patients with a *TSC1* mutation (2, 3). In this case, given the concern for a possible malignant neoplasm, further examinations were carried out with enhanced abdominal CT and bone scan after the large pelvic mass was detected. While neither renal cell carcinoma nor pancreatic neuroendocrine tumor constitutes the TSC diagnostic criteria, they are included in the updated TSC surveillance guidelines (6). Cases in which malignant tumors were detected incidentally in patients with TSC during surveillance have been reported occasionally. A misdiagnosis could relatively easily occur in TSC patients with large masses similar to our patient, particularly in those whose CT or bone scan indicates the potential for malignant neoplasm. Consequently, in the face of such patients, clinicians are supposed to strengthen their ability to distinguish benign and malignant space-occupying lesions to avoid both overdiagnosis and missed diagnosis.

SBLs, classically considered hamartomatous lesions, are frequent manifestations associated with TSC. They are usually small and multiple, more prevalent in the spine and sacrum, and are detected on chest CT, plain films, and MRI (8, 9). With the increasing recognition of SBLs, they have been included in the minor diagnostic criteria and may serve as a potential imaging biomarker for TSC (6, 10). The lesions should be differentiated from osteoblastic metastasis, particularly when patients have a history of malignancy. Previous reports, which described normal bone scintigraphy results in established sclerotic bone lesions, have suggested that bone scintigraphy may be useful in differentiating TSC from bone metastases (11–13). However, it has also been reported that bone scans showed multiple areas of increased uptake over the sclerotic bone lesions, as observed in our patient (Table 3) (14, 15). There is a possibility that bone metabolic activity is higher in these lesions in young patients than in elderly patients. In conclusion, SBLs in TSC showing abnormal uptake in bone scintigraphy should prompt consideration of benign lesions, particularly in young patients; such findings do not constitute evidence of malignancy.

**Table 3:** Previously reported cases of bone scintigraphy used in bone lesions related to TSC.

Case	Author, year	Age/sex	Malignant tumor	Radiotracer	Results
1	1996, Pui (12)	72/F	Bronchogenic carcinoma	Gd-DTPA	Bone scintigraphy showed normal uptake in the bone lesions related to TSC.
2	2001, Jonard (15)	25/F	Oncocytic adenocarcinoma	^99m^Tc-HDP	Bone scintigraphy showed heterogeneous tracer uptake in the bone lesions related to TSC.
3	2013, Brakemeier (11)	64/F	Pulmonary adenocarcinoma	^99m^Tc-MDP	Bone scintigraphy showed normal uptake.
4	2015, Parida (14)	15/M	Renal cell carcinoma	^99m^Tc-MDP	Bone lesions showed increased radiotracer uptake in the bone lesions related to TSC. SPECT/CT showed sclerosis in the same setting.
5	2023, Kumamoto (13)	52/F	Ureteral cancer	^99m^Tc-MDP	Bone scintigraphy showed no abnormalities in bone metabolism at the time of the initial diagnosis. After two courses of cisplatin, it showed increased uptake.

M, male; F, female; SPECT/CT, single-photon emission computed tomography/computed tomography.

## 4 Conclusion

We present the case of a TSC patient with an ovarian endometrioma initially suspected to represent multiple metastases because the CT and bone scintigraphy suggested the possibility of malignant neoplasm. This case report aims to enhance understanding of TSC patients with SBLs and large benign masses. As SBLs associated with TSC may show abnormal uptake on bone scintigraphy in young patients, physicians should be aware of the manifestations associated with TSC and improve their ability to differentiate between benign and malignant lesions to reduce the rate of missed diagnosis and misdiagnosis.

## Data Availability

The original contributions presented in the study are included in the article/Supplementary material, further inquiries can be directed to the corresponding authors.
