# Supplementary material for: Tuberous sclerosis complex with ovarian endometrioma misdiagnosed as multiple metastases: a case report
Source: Front Med (Lausanne). 2026 May 19;13:1819173. doi: 10.3389/fmed.2026.1819173 (PMC13226466; doi:10.3389/fmed.2026.1819173)
Supplement: Supplementary file 1 [file Data_Sheet_1.pdf]

Supplementary Material

1.1 Supplementary Figures

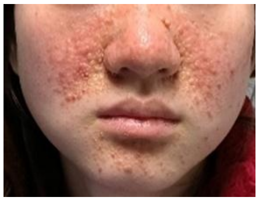

Supplementary Figure 1 Multiple facial fibromas are observed in the bilateral nasal regions.

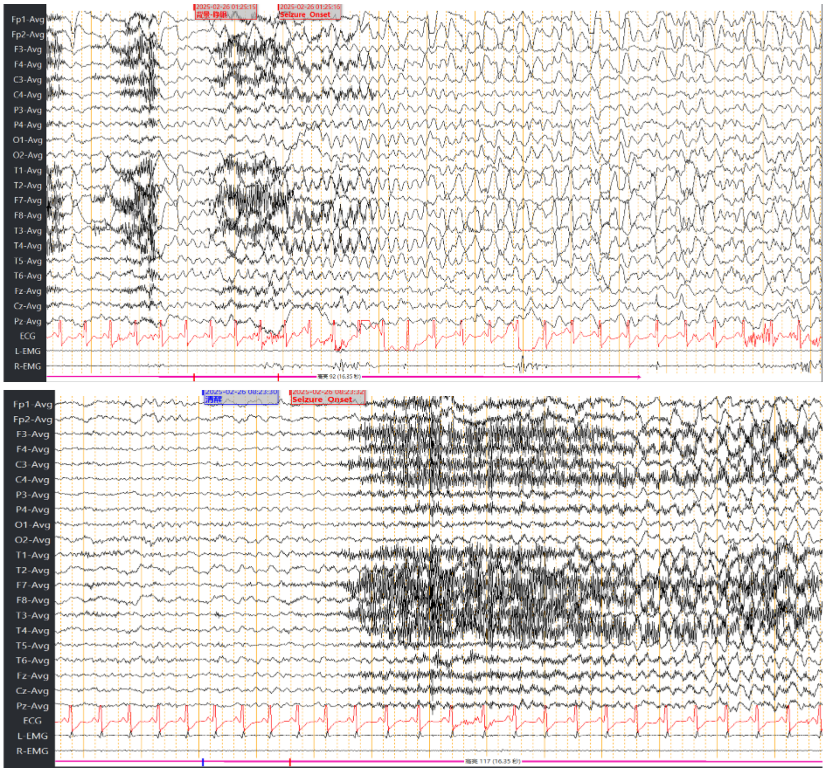

Supplementary Figure 2 Electroencephalography showed multiple epileptic discharges.
